# Metabolome and Transcriptome Integration Reveals Insights Into Flavor Formation of ‘Crimson’ Watermelon Flesh During Fruit Development

**DOI:** 10.3389/fpls.2021.629361

**Published:** 2021-05-12

**Authors:** Chengsheng Gong, Weinan Diao, Hongju Zhu, Muhammad Jawad Umer, Shengjie Zhao, Nan He, Xuqiang Lu, Pingli Yuan, Muhammad Anees, Dongdong Yang, M. O. Kaseb, Wenge Liu

**Affiliations:** Zhengzhou Fruit Research Institute, Chinese Academy of Agricultural Sciences, Zhengzhou, China

**Keywords:** watermelon, transcriptome, metabolome, sugars, volatile organic compounds, coexpression

## Abstract

Metabolites have been reported as the main factor that influences the fruit flavor of watermelon. But the comprehensive study on the dynamics of metabolites during the development of watermelon fruit is not up-to-date. In this study, metabolome and transcriptome datasets of ‘Crimson’ watermelon fruit at four key developmental stages were generated. A total of 517 metabolites were detected by ultrahigh-performance liquid chromatography–electrospray ionization–tandem mass spectrometry and gas chromatography–solid-phase microextraction–mass spectrometry. Meanwhile, by K-means clustering analysis, the total differentially expressed genes were clustered in six classes. Integrating transcriptome and metabolome data revealed similar expression trends of sugars and genes involved in the glycolytic pathway, providing molecular insights into the formation of taste during fruit development. Furthermore, through coexpression analysis, we identified five differentially expressed ADH (alcohol dehydrogenase) genes (*Cla97C01G013600*, *Cla97C05G089700*, *Cla97C01G001290*, *Cla97C05G095170*, and *Cla97C06G118330*), which were found to be closely related to C9 alcohols/aldehydes, providing information for the formation of fruit aroma. Our findings establish a metabolic profile during watermelon fruit development and provide insights into flavor formation.

## Introduction

Flavor quality is one of the most important criteria for consumers to select vegetables and fruits (Barrett et al., 2010) and promotes customers’ desire for reconsumption. However, as flavor phenotyping is complex, expensive, and not suitable for high-throughput analysis, most breeders tend to focus more on yield, appearance quality, and plant resistance than fruit flavor quality (Tieman et al., 2017). The flavor is typically described as a sum of the interactions between taste and aroma. Generally speaking, the formation of flavor is the result of the interaction of multiple metabolites. The taste is activated mainly through taste receptors in the mouth. Sugars and acids are an essential basis for evaluating the taste of fruits such as tomatoes (Zhu et al., 2018). The aroma is the sense of smell through the nose to perceive volatile organic compounds (VOCs). Different fruits have different aromas that may result from a combination of multiple metabolites, for instance, 2,6-nonadienal is the characteristic VOC in cucumber (Forss et al., 1962), but it is also detected in other fruits with different fruit aromas. As the market demand for high-quality fruit is a feature of modern consumer society, a more comprehensive understanding of fruit flavor-related metabolites and their improvement should be an important objective in breeding programs (Kyriacou and Rouphael, 2018).

Watermelon (*Citrullus lanatus*) is among the top five most consumed fresh fruits globally, with unique flavor, rich nutrition, and health benefits to humans (Guo et al., 2013; Zhang et al., 2017). Ramirez et al. (2020) conducted a sensory profile of seven watermelon varieties, and the result indicates that flavor had an important effect on the refreshing perception of watermelon. Undoubtedly, the formation of watermelon flavor is a combination of different metabolites, although the current understanding of flavor metabolites is still insufficient. Sweetness is a crucial factor for the commercial value of watermelon, but studies on sweetness mainly focus on sucrose, fructose, and glucose (Gao et al., 2018b), and research on watermelon acidity has focused on malic acid, citric acid, and oxalic acid (Umer et al., 2020). In parallel, although the acidity in watermelon is relatively low, it may regulate the feeling of sweetness (Soteriou et al., 2014). Progress has been made in watermelon breeding by improving metabolites. Gao et al. (2018a) developed a new watermelon variety ‘SW’ with a sweet and sour taste, which is an innovation in the breeding of watermelon taste. VOCs play a key role in the formation of fruit flavor, Bianchi et al. (2020) detected a total of 20 VOCs at the ripening stage of two mini watermelons ‘Rugby’ and ‘Cuoredolce^®^,’ of which 13 were C9 aldehydes and alcohols [a metabolite with nine C atoms in the lipoxygenase (LOX) pathway, such as 2,6-nonadienal, (E,Z)-]. Existing studies have promoted the study of watermelon flavor; however, it is necessary to conduct a comprehensive and extensive metabolomic study on the development process of watermelon to establish watermelon flavor. Related research has been found in melon and other fruits; for example, approximately 2,000 metabolite signatures and 15 mineral elements were detected in melon, which provided valuable information for the study of melon flavor and nutrition (Moing et al., 2011). In recent years, with the continuous improvement of detection technology, the widely targeted metabonomics method developed by Chen et al. (2013) can realize the high-throughput determination of metabolites, and solid-phase microextraction (SPME)–gas chromatography (GC)–MS has been widely used in the detection of VOCs. The progress of detection methods provides a powerful tool for us to carry out watermelon flavor research.

Transcriptional regulation is the most important regulation mode of organisms. With the continuous development of sequencing technology, transcriptome has been widely used. For instance, Guo et al. (2015) systematically explained the molecular basis of differences in important traits, such as sugar content, flesh texture, and color through transcriptome profiles analysis of wild and cultivated watermelon; Gao et al. (2018b) identified the key genes of soluble sugars and organic acids through integrated analysis in a subsequent study. Notably, integrating of transcriptome data with metabolic profiles is an effective method to parse metabolic pathways and plant gene function (Luo, 2015); however, related research in watermelon is extremely lacking. In plants, metabolites associated with taste are synthesized and degraded through critical biological processes such as glycolysis and the tricarboxylic acid cycle (Nativ et al., 2017). VOCs are synthesized mainly through methylerythritol phosphate or mevalonate pathway to generate terpenoids; phenylpropane/benzene compounds through aromatic amino acids; and C6 and C9 alcohols/aldehydes formed from unsaturated fatty acids, such as linoleic acid and linolenic acid (Dudareva et al., 2013; Wei et al., 2016); the study of metabolic pathways has increased the understanding of the links between genes and metabolites. In conclusion, data integration platform is a helpful approach to exploring the hidden diversity in flavor formation by using transcriptome and metabolome profiles.

In this study, we generated the metabolome and transcriptome datasets from the ‘Crimson’ watermelon cultivar at 10, 18, 26, and 34 days after pollination (DAP). ‘Crimson’ watermelon cultivar is famous for its nutritionally rich flavor and is widely used in breeding programs. Ultrahigh-performance liquid chromatography–electrospray ionization–tandem mass spectrometry (UPLC–ESI–MS/MS) and GC–SPME–MS detected a total of 517 metabolites. The accumulation patterns of flavor-related metabolites, such as sugars, organic acids, and VOCs, were analyzed. The differentially expressed genes (DEGs) were clustered into six classes by K-mean clustering analysis. Furthermore, the metabolites and genes related to flavor enriched into related metabolic pathways were identified, which provides more comprehensive information for the formation of flavor during the development of watermelon fruit. This study provides molecular insights into the metabolites associated with formation of watermelon flesh flavor and lays a foundation for further studies on metabolomics.

## Materials and Methods

### Experimental Material and Sampling

The experimental materials, watermelon cultivar ‘Crimson’ seeds, were collected from the National Mid-term Genebank for Watermelon and Melon, Zhengzhou Fruit Research Institute, Chinese Academy of Agricultural Sciences (Zhengzhou, Henan, China). ‘Crimson’ has a spherical fruit with red color flesh, resistance to disease, nutritionally rich taste, and aroma. Plants of watermelon were planted in the greenhouse (Xinxiang, 35°18′ N, 113°55′ E) in Henan Province, China. All the standard agronomic practices were followed during the developments, such as irrigation, weeding, and fertilization. The seedling was raised and transplanted to the field on April 13, 2019. The experimental design was a completely randomized block design and plant–plant spacing of 0.8 m and a row–row spacing of 1.5 m. And pollination work starts on May 11, 2019. After transplanting, the temperature and humidity of the greenhouse were artificially adjusted: in the daytime, the temperature was generally 25–35°C, and the humidity was 55–70%; in the evening, the temperature was generally more than 15°C, and the humidity was 75–80%. Pollination was done manually: every a second female flower was artificially self-pollinated and tagged the pollination date, only one watermelon fruit allowed to grow on one plant. Flesh samples were preserved immediately in liquid nitrogen; from all four key developmental stages 10 DAP (immature white), 18 DAP (white-pink), 26 DAP (red), and 34 DAP (ripe) (Zhang et al., 2017), the samples were represented by KL1 (10 DAP), KL2 (18 DAP), KL3 (26 DAP), and KL4 (34 DAP), respectively. The well-grown watermelon fruit, uniform in appearance and without visual defects, was cut in the longitudinal direction, and the center flesh was taken, avoiding seeds. Three biological replicates were collected for each stage, and a total of 12 samples were collected. All the flesh samples were grounded in powder and preserved in liquid nitrogen for further transcriptome and metabolome analysis. A balanced mixture of 12 collected samples was used as the mixed sample for quality control.

### Measurement of Fruit Weight, Flesh Firmness, Total Soluble Solids Content, and pH of Watermelon Flesh

At four key development stages, watermelon fruit weight was measured by an electronic scale. When the fruit was cut lengthwise, the center firmness of flesh was determined by FT 011 fruit firmness tester. And after the center flesh was homogenized, a laboratory refractometer (HC-112ATC, Shanghai LICHENKEYI, China) and a PHB-4 (Shanghai LICHENKEYI, China) instrument was used to determine total soluble solids content (TSS, Brix%) and pH, respectively (Gao et al., 2018b). Three separate well-grown fruits were measured and recorded at each developmental stage. The taste of watermelon was evaluated by human mouth and the nose to perceive the aroma of the fruit.

### LC–ESI–MS/MS System-Based Widely Targeted Metabolomics Analysis

Freeze-dried watermelon flesh samples were grided evenly by using a mixer mill (MM 400, Retsch) for 1.5 min at 30 Hz. The preparation of watermelon fruit extract was performed as previously described (Qin et al., 2020). In brief, 100-mg sample powder was extracted overnight with 1.2 mL 70% methanol solution at 4°C. After centrifugation at 10,000 *g* for 10 min, the extracts were absorbed (CNWBond Carbon-GCB SPE Cartridge; Anpel, Shanghai, China) and filtered with an SCAA-104 membrane (Anpel, Shanghai, China) for further UPLC–MS/MS analysis.

UPLC–ESI–MS/MS system (UPLC, Shim-pack UFLC SHIMADZU CBM30A system; MS, Applied Biosystems 6500 Q TRAP) was used to analyze the sample extracts. The analytical conditions were as follows: chromatographic column, Waters ACQUITY UPLC HSS T3 C18 (1.8 μm, 2.1 × 100 mm). The column temperature was 40°C, the injection volume was 2 μL, and the procedure for sample measurements by UPLC was as follows: the initial condition was that 95% solvent A (pure water with 0.04% acetic acid), 5% solvent B (acetonitrile with 0.04% acetic acid); after 10 min, the linear gradient to 5% solvent A and 95% solvent B was programmed and kept 1 min, and then solvent B changed to 5% in 0.1 min and kept for 2.9 min, with a flow rate of 0.35 mL/min. Triple quadrupole-linear ion trap mass spectrometer (Q TRAP), equipped with an ESI turbo ion-spray interface, ran in positive and negative ion mode, and was controlled by analyst 1.6.3 software (AB SCIEX). The operating parameters of the ESI were as follows: source temperature was 550°C, ion spray voltage was 5,500 V in positive ion mode and −4,500 V in negative ion mode, and curtain gas (CUR) was set at 50, 60, and 30.0 psi, respectively, and the collision gas (CAD) was high. The instrument was tuned and mass calibrated with 10 and 100 μmol/L propanediol solutions in QQQ and LIT modes, respectively. QQQ scans were used as MRM experiments, and the collision gas (nitrogen) was set to as 5 psi. Further declustering potential and collision energy optimizations were performed for individual MRM transitions. Based on the metabolites eluted during this period, a specific set of MRM transitions was monitored in each period.

Qualitative analysis of MS data was based on the comparison of the retention time (RTS) and fragmentation pattern, accurate precursor ion (Q1) value, product ion (Q3) value, data of which were obtained the from standard injection under the same conditions (Sigma–Aldrich, United States); if no standard, the analysis was performed using the MWDB database (Metware Biotechnology Science and Technology Co., Ltd.), the Kyoto Encyclopedia of Genes and Genomes (KEGG) database^1^, and the publicly available metabolite databases online at PLANTCYC^2^, etc. MRM mode was used for quantitative analysis. The characteristic ions of each metabolite were screened by QQQ MS, and the signal intensity was obtained. The chromatographic peaks were integrated and corrected using Multiquant version 3.0.2 (AB SCIEX, Concord, Canada). The corresponding relative metabolite content was expressed by chromatographic peak area integral. In addition, metaX software^3^ was used for screening and quantitative analysis of the differences of metabolites.

### Extraction of Volatiles and SPME–GC–MS Analysis

Fresh samples were gridded into fine powder in liquid nitrogen, and 1 g of powder was immediately transferred to a 20-mL headspace vial (Agilent, Palo Alto, CA, United States) with NaCl saturated solution. The vials were sealed using crimp-top with TFE-silicone headspace septa (Agilent). During SPME analysis, each vial was placed at 60°C for 10 min. A 50/30 μm divinylbenzene/carboxen/polydimethylsilioxane fiber (Sigma) was exposed to the sample’s headspace for 20 min at the temperature of 60°C. After sampling, desorption was performed at 250°C for 5 min at the injection port of the GC apparatus (Model 7890B; Agilent) in a shunt-free mode. The model of the mass spectrum was 7000D (Agilent); capillary column: 30 m × 0.25 mm × 0.25 μm DB-5 ms (5% phenyl polymethylsiloxane); carrier gas: helium, with a linear velocity of 1.0 mL/min; injector temperature: 250°C; and detector temperature: 280°C. The oven temperature was programmed from 40°C for 5 min and increased at 6°C/min to 280°C for 5 min; electron impact ionization mode: 70 eV; quadrupole mass detector temperature: 150°C; ion source temperature: 230°C; transfer line temperature: 280°C. The scanning range of MS was m/z 50–500 AMU, and the interval was 1 s. The volatile compounds were identified by comparing MS with the data system library (MWGC or NIST) and linear retention index.

### Constriction of RNA-Sequencing Libraries

The total RNA was extracted by RNA extraction kit (TIANGEN, Beijing, Biotech, China) following the manufacturer’s instructions. Furthermore, 1% agarose gels electrophoresis was used to analyze the integrity of RNA and any other contamination. The purity of RNA was detected by Nanodrop Nano Photometer (IMPLEN, GmbH, Munich, Germany); RNA concentration was measured by Qubit 2.0 Fluorometer, and the integrity of RNA was checked accurately by Agilent 2100 biological analyzer. Sequencing libraries were established using the NEBNext^®^ Ultra^TM^ RNA Library Prep Kit for Illumina^®^ (NEB, United States) and sequenced on an Illumina HiSeq platform, and 125-bp/150-bp paired-end reads were generated. After the libraries’ detection, the sequencing was performed on the Illumina HiSeq platform. Raw reads were filtered to get good quality reads. Data were thoroughly checked to identify any false reads; moreover, GC content was also monitored, so that clean reads were obtained and can be used for the subsequent steps. Hisat2 was used to sequence clean reads with the reference genome (97103 V2)^4^. Transcripts or gene expression levels were measured by calculating FPKM (fragments per kilobase of transcript per million fragments mapped) (Mortazavi et al., 2008). Deseq2 (Love et al., 2014; Varet et al., 2016) was used to analyze differential gene expression among different samples. After that, multiple hypothesis tests were used to correct the hypothesis test probability (*P*-value) by Benjamin Hochberg to obtain the false discovery rate (FDR), and the Bioconductor package CusterProfller was used for KEGG enrichment analysis.

### Differential Metabolites and Genes Analysis

For the analysis of differentially accumulated metabolites (DAMs), the two developmental stages’ screening criteria were as follows: (1) The metabolite accumulation levels were that fold change ≥ 2 or fold change ≤ 0.5; (2) based on the OPLS-DA model analysis results, metabolites with VIP (variable importance in project) ≥ 1 (Yuan et al., 2018). The relative contents of differential metabolites were standardized and centralized before analysis. For DEGs between two developmental stages, the screening criteria were | log_2_fold change| ≥ 1 and FDR < 0.05. Principal component analysis (PCA) diagram, hierarchical clustering analysis diagram, and Venn diagram were drawn by R software package, and the Pearson correlation coefficient (PCC) of genes and metabolites was calculated by the COR program in R. Coexpression analysis network diagram drawn by Cytoscape software (version 3.7.1) (Kohl et al., 2011).

## Results

### The Ripeness of the Watermelon Fruit Is Adapted to the Formation of the Flavor

**FIGURE 1:**
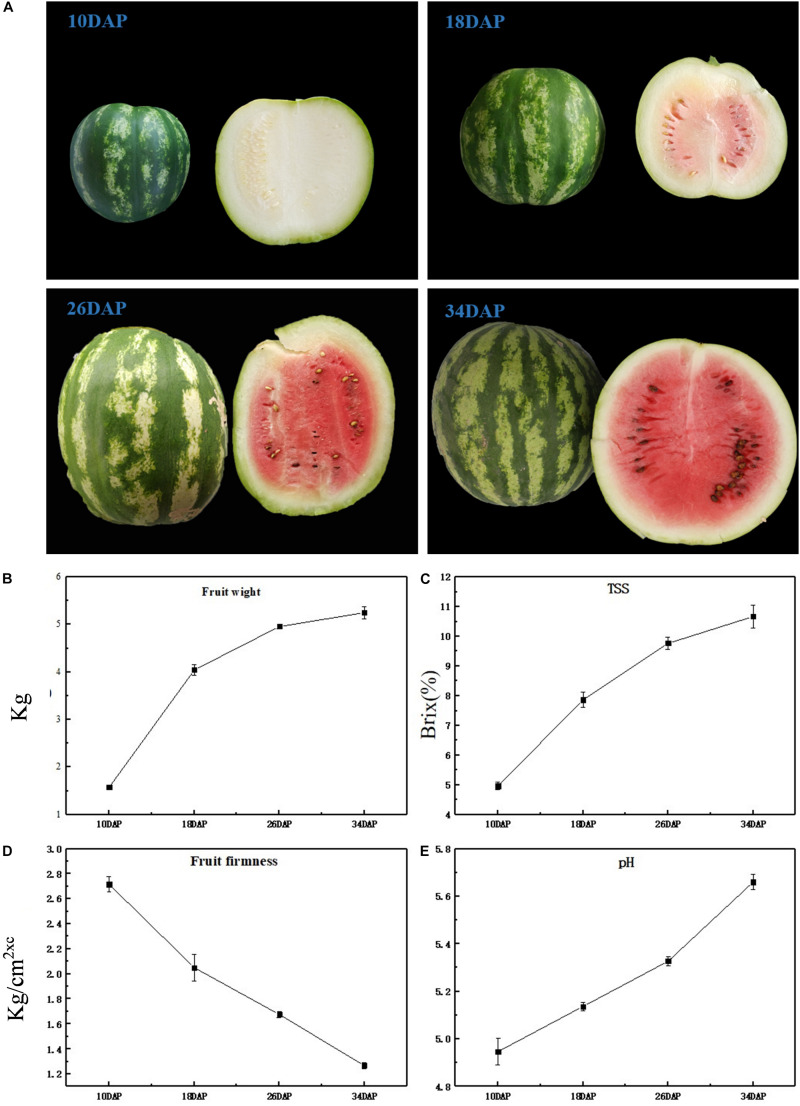
Physiological characteristics of ‘Crimson’ watermelon fruit. **(A)** The appearance of ‘Crimson’ at four key development stages (10, 18, 26, and 34 DAP). **(B–E)** Fruit weight, TSS content (Brix%), flesh firmness, and pH during different development stages of ‘Crimson’ watermelon fruit, respectively. And each stage of these four agronomic traits took three biological replicates.

The most notable difference was that as watermelon fruits develop and ripen, the color of the flesh changes from light white to bright red (Figure 1A); at the same time, through taste evaluation, it was found that at 10 DAP, the taste of watermelon fruit was light, but at 34 DAP, the taste of watermelon became sweet and delicious; this corresponds to olfactory perception; at 10 DAP, the intensity of aroma was not much pleasant, but with a fresh and pleasant aroma at 34 DAP. Notably, significant phenotypic variation was obtained. Average fruit weight increased rapidly from 1.57 kg at 10 DAP to 4.04 kg at 18 DAP, and no significant difference was observed from 26 to 34 DAP (Figure 1B). An increasing trend was observed for TSS content at all development stages, i.e., 4.97, 7.87, 9.77, and 10.67 at 10, 18, 26, and 34 DAP (Figure 1C), respectively, whereas watermelon flesh’s firmness was measured as 2.72, 2.05, 1.68, and 1.27 kg/cm^2^ at 10, 18, 26, and 34 DAP (Figure 1D), which has an opposite trend compared with fruit weight and TSS. pH can reflect the change of watermelon acidity, high pH is consistent with low acidity. pH values were recorded as 4.95, 5.14, 5.33, and 5.66 at 10, 18, 26, and 34 DAP (Figure 1E), respectively, indicating a decrease in acidity during fruit development. As the fruit grows heavier and softer during watermelon development and ripening, the taste and aroma of the fruit become better. Changes of TSS and pH correspond to an increase in sweetness and a decrease in acidity, often caused by metabolite changes.

### Overview of Metabolite Accumulation Patterns During Watermelon Fruit Development and Analysis of Key Metabolites Related to Taste

**FIGURE 2:**
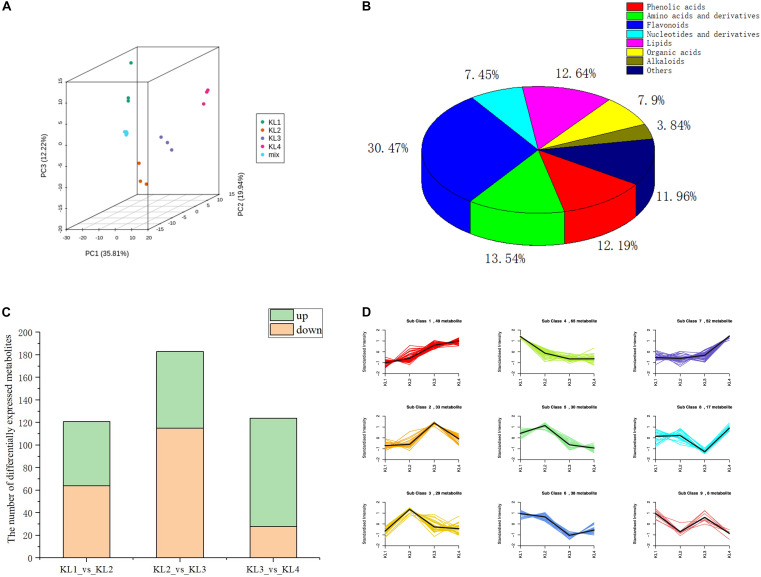
The analysis results of the metabolic profiles obtained by LC–MS during the development of watermelon fruit. **(A)** Principal component analysis (PCA) score plot of all metabolites in 12 samples. KL1, KL2, KL3, and KL4 represent the samples at 10, 18, 26, and 34 days after pollination, respectively (DAP). The samples with the same color represent three biological replications of the same period. **(B)** Pie chart of the classification of 443 metabolites. **(C)** The number of differentially accumulation metabolites (DAMs) by comparing KL1 vs. KL2, KL2 vs. KL3, and KL3 vs. KL4. Green represents the number of up-regulated metabolites, On the contrary, orange stands for down-regulation. **(D)** Dynamics of Metabolite during four development stages. The bold black line represents the average pattern of all candidate metabolites in each class; the different colored lines represent different classes, and each line represents the average relative value of the metabolite over three biological replicates.

With the development of watermelon fruit, a variety of metabolites were synthesized and degraded. As taste results from the interaction of multiple metabolites, enhancing the information of metabolite changes is conducive to a more comprehensive understanding of watermelon taste formation. Therefore, we have carried out a widely targeted metabolomics method to detect metabolites to recognize metabolite accumulation patterns on a global level. A total of 443 metabolites were detected at different developmental stages, i.e., 10, 18, 26, and 34 DAP by UPLC–ESI–MS/MS. The PCA results showed that the first three principal components explain 35.81, 19.94, and 12.22% of the samples’ variance, respectively (Figure 2A), and the heat map of metabolites can clearly distinguish the watermelon samples at different developmental stages (Supplementary Figure 1). These metabolites contain 135 flavonoids, 60 amino acids and derivatives, 56 lipids, 54 phenolic acids, 53 others, 35 organic acids, 33 nucleotides and derivatives, and 17 alkaloids (Figure 2B and Supplementary Table 1), which shows the diversity of metabolites during watermelon fruit flesh development.

To further clarify the accumulation pattern of different metabolites during watermelon fruit development, K-means clustering algorithm was performed. Three hundred twenty-one DAMs were obtained by comparing the metabolites at different developmental stages, and 121, 183, and 124 DAMs were screened by comparing KL1 (10 DAP) vs. KL2 (18 DAP), KL2 vs. KL3, and KL3 (26 DAP) vs. KL4 (34 DAP), respectively (Supplementary Table 2). Significantly, KL2 vs. KL3 had the most significant down-regulated metabolites (115), and KL3 vs. KL4 had the largest number of up-regulated metabolites (96) (Figure 2C), indicating that there was a more active metabolic activity from 18 to 34 DAP, which is the key period of watermelon flavor formation. Nine classes were obtained through the analysis of all the DAMs, among which the content of candidate metabolites in classes 1 and 7 presented an increasing trend, with 49 and 52 DAMs, respectively, such as sucrose citrulline and histidine; in contrast, 65 candidate DAMs in class 4 showed a decreasing trend, similarly, in classes 5 and 6, the metabolites also showed a downward trend during the watermelon flesh development, and there were 30 and 38 candidate DAMs, such as naringenin chalcone, vanillin, salicylic acid, and adenosine (Figure 2D and Supplementary Table 1). Watermelon flavor is derived from the interaction of multiple metabolites, and the watermelon fruit has better flavor during its development, so the metabolites in classes 1 and 7 can be used as potential flavor-related metabolites.

Sugars and organic acids play an important role in the taste formation of watermelon fruits. The K-means cluster analysis included 9 different sugars and 21 different organic acids. Among them, all nine sugars were enriched in class 1 (Supplementary Table 1), such as glucose, trehaloseanhydrous, trehalose 6-phosphate, and sucrose, and showed the same trend with TSS, indicating that these sugars were accumulated during the development of fruit and were responsible for the sweetness of the mature watermelon. The 11 organic acids, including shikimic acid and citridic acid, have a negative accumulation trend and were enriched in classes 4, 5, and 6, which may be adapted to the increase of pH during watermelon fruit development. These key metabolites are thought to be related to the formation of watermelon taste.

As fatty acids are important prerequisites for the generation of alcohols/aldehydes and other VOCs, we analyze the accumulation pattern of fatty acids here to increase our understanding of the synthesis of aroma-related metabolites. It is worth noting that of 16 differential free fatty acids, all but pentadecanoic acids were enriched in class 4, suggesting that the content of free fatty acids decreases during the development of watermelon fruits. However, the aroma of watermelon increased during ripening, so the degradation of free fatty acids was considered related to the synthesis of VOCs. Subsequently, VOCs, especially the metabolites of the LOX pathway, were further determined to understand the formation of watermelon aroma during fruit development.

### Eleven of the 74 VOCs Were Significantly Related to the Formation of Aroma During the Development of Watermelon Fruit

**FIGURE 3:**
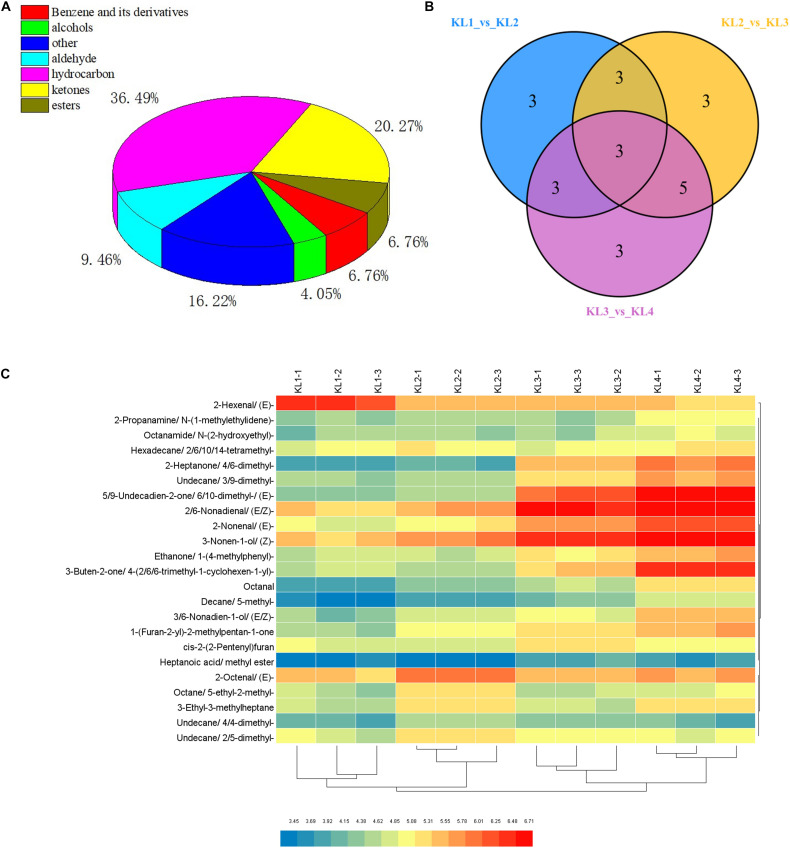
Classification of volatile organic compounds (VOCs) detected by GC–MS and analysis of differential metabolites. **(A)** Pie chart of the classification of 74 VOCs. **(B)** Venn diagram of the number of differential accumulation VOCs by comparing KL1 vs. KL2, KL2 vs. KL3, and KL3 vs. KL4. **(C)** Hierarchical clustering heat map of 23 differential accumulation VOCs in 12 samples, KL1, KL2, KL3, and KL4 represent the samples at 10, 18, 26, and 34 DAP, and -1, -2, and -3 represent different samples from the same period.

To provide insights into the formation of watermelon flesh aroma, VOCs were measured by GC–SPME–MS. Here, a total of 74 VOCs were detected, including 27 hydrocarbons, 15 ketones, 7 aldehydes, 5 benzene and its derivatives, 5 esters, 3 alcohols, and 12 others (Figure 3A and Supplementary Table 3). By comparing KL1 vs. KL2, KL2 vs. KL3, and KL3 vs. KL4, a total of 23 differentially accumulated VOCs were detected, and there were 12 (11 up-regulated and 1 down-regulated), 14 (11 up-regulated and 3 down-regulated), and 14 metabolites (all up-regulated), respectively (Figure 3B and Supplementary Table 4), among which, decane, 5-methyl-; 2-nonenal, (E)-; and 3-ethyl-3-methylheptane were the different common metabolites.

Furthermore, hierarchical clustering analysis showed that the accumulation of DAMs was developmental stage-specific (Figure 3C). It can be clearly observed that 2-hexenal, (E)- has a higher accumulation at 10 DAP, whereas 2-heptanone, 4,6-dimethyl-; undecane, 3,9-dimethyl-; 5,9-undecadien-2-one, 6,10- dimethyl-,(E)-; 2,6-nonadienal,(E,Z)-; 2-nonenal, (E)-; 3-nonen-1-ol, (Z)-; ethanone, 1-(4-methylphenyl)-; 3-buten-2-one,4-(2,6,6-trimethyl-1-cyclohexen-1-yl)-; 3,6-nonadien-1-ol, (E,Z)-; and 1-(Furan-2-yl)-2-methylpentan-1-one were significantly enriched at 34 DAP. These 11 VOCs were defined as the top abundant components of aroma. Interestingly, we found that the important VOCs such as 2-hexenal, (E)-; 3,6-nonadien-1-ol, (E,Z)-; 2,6-nonadienal, (E,Z)-; 3-nonen-1-ol, (Z)-; and 2-nonenal, (E)-, which were synthesized by the LOX pathway, were significantly enriched in different development stages.

### K-Means Clustering Analysis Divides the Expression Pattern of DEGs Into Six Classes

To further investigate changes in gene expression during watermelon fruit at four developmental stages, transcriptome sequencing was performed on 12 samples, and a total of 98.22-Gb clean data were obtained. The clean data of each sample reached six Gb, and the Q30 base percentage was 90% or greater. FPKM was calculated as an index to measure the expression level of transcripts or genes (Supplementary Table 5). Correlation analysis was conducted by calculating PCC, and the results showed that for the three samples in the same period, *R*^2^ was all greater than 0.9 (Supplementary Figure 2A), indicating good reproducibility between test samples. Overall, the obtained sequencing data were of high quality and could be performed for further analysis. The transcriptome data of watermelon flesh at different developmental stages were separated by PCA, and PC1 and PC2 were 45.63 and 16.6%, respectively (Supplementary Figure 2B). These results suggest that our samples have good reproducibility, and there are significant differences in gene expression levels of samples in different development stages.

Different genes in an organism perform different biological functions through interaction, and the pathway annotation analysis of DEGs helps to further interpret the functions of genes. Through KEGG annotation, it was found that the most abundant KEGG terms were metabolic pathways and biosynthesis of secondary metabolites (Supplementary Figure 3), implying that genes were involved in a variety of metabolic activities during watermelon fruit development.

Similar to metabolite analysis results, hierarchical clustering analysis showed that DEGs were characterized by specific expression at different developmental stages of watermelon fruit (Supplementary Figure 4A), suggesting a potential relationship between genes and metabolites. To understand gene expression patterns during watermelon fruit development, the K-means clustering algorithm was used. K-means cluster analysis results showed that DEGs were divided into six classes, from 10 DAP to 34 DAP (Supplementary Figure 4B). Genes of classes 1 and 2 have 2,376 and 3,199 genes, respectively, and showed a downward expression trend, and these genes may play a regulatory role in metabolite accumulation during fruit development; conversely, the DEGs of class 4 (1,549) and class 5 (1,531) was up-regulated during watermelon development, as metabolites associated with flavor formation are positively accumulated during the developmental process of watermelon; therefore, these genes may play an important role in regulating the metabolites associated with flavor-related metabolites. Transcription factors (TFs) may regulate the transcription of multiple genes during gene regulation; as shown here, it is worth noting that by comparing KL1 vs. KL2, KL2 vs. KL3, and KL3 vs. KL4, we found 300, 462, and 258 TFs were identified, respectively. Furthermore, the statistics show that there were 162, 271, 86, 48, 80, and 54 TFs with differential expression from class 1 to class 6 (Supplementary Figure 4C). The same expression trend was found in TFs and all genes, suggesting that TFs play an important role in regulating structural genes during watermelon fruit development. Similarly, genes with the same or opposite accumulation patterns are more likely to regulate the accumulation of metabolites.

### Integrating Related Genes and Metabolites in the Glycolytic Pathway Provides Molecular Insights Into the Formation of Sweet Taste

**FIGURE 4:**
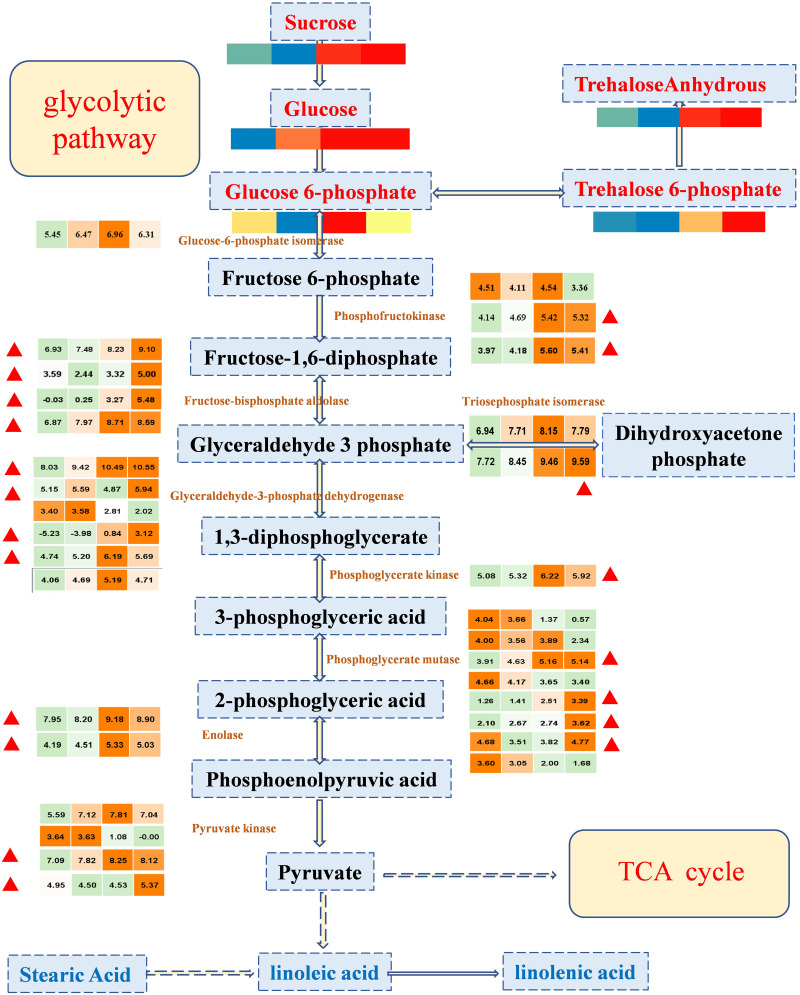
The expression profile of important regulatory genes and the accumulation patterns of sugars in the glycolytic pathway during four important developmental periods. In the color scale, each cube metabolized the average of three replicates in the same period. The metabolite data were standardized by the logarithm of 10, and the gene was the logarithm of 2; red or orange represents a higher relative content, but blue or green represents a lower relative content, from left to right represents the average relative content of metabolites at 10, 18, 26, and 34 DAP, respectively. The red triangle represents that the enriched genes in class 4 and class 5 are in the K-means clustering analysis, showing a tendency of up-regulation.

Sweetness is the most important taste in fruit, and it is the most attractive characteristic of the cultivated watermelon fruit flesh (Guo et al., 2015). The synthesis and degradation of sugars are usually dependent on glycolytic pathways, which provide energy for living organisms and provide substrates to synthesize many metabolites. To explore the potential links between the metabolome and the transcriptome data, sugars and DEGs were allocated to glycolysis pathways (Figure 4). Through data integration analysis, for genes, we observed that important genes in glycolytic pathway were highly expressed in 26 and 34 DAP. Most of these genes were enriched in classes 4 and 5 (with up-regulated expression trend, marked in Figure 4), suggesting that vigorous metabolic activities were carried out during fruit development; sugars, such as glucose and sucrose, positively accumulated with fruit development. As the expression of genes and the accumulation of sugars have the same trend, the accumulation of sugars may be mainly the regulation from the transcriptional level. In conclusion, sugars provide substrates for the active development of glycolytic pathway, and the positive expression of glycolytic pathway genes also promotes the accumulation of sugars; the formation of sweet taste in watermelon fruit was relatively consistent with the vigorous gene activity.

### Coexpression Analysis of Lipid Metabolites Related Genes in LOX Pathway Provided Insights Into the Formation of Watermelon Aroma

C9 alcohols and aldehydes produced by the LOX pathway are important aroma metabolite components in watermelon development. LOX pathway uses linolenic acid as the precursor to synthesizing related VOCs under the regulation of a series of genes. LOX and ADH (alcohol dehydrogenase) have been shown to play a key role in the synthesis of alcohols and aldehydes (Dudareva et al., 2013).

**FIGURE 5:**
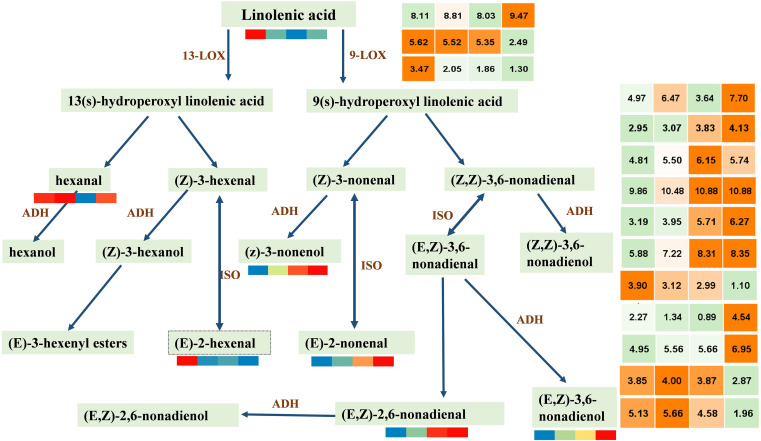
Accumulation patterns of C6 and C9 alcohols/aldehydes synthesized by lipoxygenase pathway and expression patterns of key genes. LOX, lipoxygenase; ADH, alcohol dehydrogenase; ISO, isomerase. In the color scale, each cube metabolized the average of three replicates in the same period. The metabolite data were standardized by the logarithm of 10, and the gene was the logarithm of 2; red and orange represent a higher relative content, and blue represents a lower relative content. From left to right represents the average relative expression of metabolites at 10, 18, 26, 34 DAP, respectively. The gene expression profile information is shown in Supplementary Table 6.

For further clarification of this study, we screened DEGs by reference genome (see text footnote 4) search combined with FPKM having a period greater than at least 10. Thus, 3 *LOX* and 11 *ADH* were screened (Figure 5). We can observe that the contents of C6 VOCs (hexanal and 2-hexenal, (E)-) are higher when the watermelon was immature, whereas the contents of C9 VOCs (2,6-nonadienal, (E,Z); 2-nonenal, (E)-; 3,6-nonadien-1-ol, (E,Z)-; and 3-nonen-1-ol (Z)-) are higher when the watermelon was mature, indicating that the C9 alcohols and aldehydes were the main aroma metabolites of watermelon. LOX (Cla97C06G115570) and eight ADHs (Cla97C09G172570, Cla97C01G013600, Cla97C05G092340, Cla97C05G089700, Cla97C01G001290, Cla97C05G095170, Cla97C08G152400, Cla97C06G118330) in class 4 or 5 showed the same trend as the C9 VOCs. It is worth noting that the results of PCC analysis between genes and metabolites showed that five ADH genes, i.e., *Cla97C01G013600*, *Cla97C05G089700*, *Cla97C01G001290*, *Cla97C05G095170*, and *Cla97C06G118330* were associated with the metabolite 2,6-nonadienal, (E,Z); 2-nonenal, (E)-; 3,6-nonadien-1-ol, (E,Z)-; and 3-nonen-1-ol, (Z)- and had a highly positive correlation (Supplementary Figure 5), which suggests that these genes may play an important regulatory role in the synthesis of metabolites. In conclusion, four key C9 aldehyde/alcohol compounds to the formation of watermelon aroma are closely related to five ADHs with the same expression trend, providing new ideas and important data on the formation of watermelon aroma.

## Discussion

Metabolome is often thought of as a ‘readout’ of physiological states (Fang et al., 2019). Therefore, it is widely used in the study of plant growth and development, biological and abiotic stress, etc. The metabolome provides an essential means for the study of the nutrition and flavor of watermelon. In previous study, 18 kinds of lycopene, 3 kinds of sugars, 5 kinds of organic acids, and 16 kinds of VOCs were detected in 12 watermelon cultivars by HPLC, GC–flame-ionization detection, and GC–MS (Liu et al., 2012), which increased the understanding of the difference of metabolites in flesh of different flesh color. Although metabolome detection technology has made rapid progress, studies on metabolites in watermelon were usually focused only on a small number of metabolites, such as sucrose, glucose, and malic acid (Gao et al., 2018b; Umer et al., 2020). And the overall understanding of the metabolic profile during watermelon fruit development remains rare. Metabolic profiles of the four developmental stages showed that the levels of 321 of the 443 metabolites differed in at least one developmental stage by LC–MS analysis. Simultaneously, 23 of 74 VOCs had differences, which were analyzed by GC–MS. Such significant changes in multiple metabolites during development have also been found in fruits such as apples (Xu et al., 2020). Here, we have built a global map of the significant metabolic changes that occur during watermelon fruit development.

Flavor is the sum of taste and aroma; moreover, sweet and sour taste is an important index to measure the taste of fruit, and VOCs can cause the production of fruit aroma. The mature watermelon had better taste, so the metabolites in classes 1 and 7 were more likely to be taste related. Sugar accumulation is an important feature of watermelon development and maturation. All the nine different accumulated sugars were enriched in class 1, which was consistent with the accumulation trend of TSS by Guo et al. (2015). Furthermore, we found that most essential amino acids such as L-tryptophan, L-proline, L-histidine, and other metabolites were also enriched in these two classes, which combined with watermelon had better taste at maturity stage. The increase in pH indicates that the acidity of watermelon decreases during the development process, which may be related to the decrease of 11 organic acids obtained by our analysis. However, malic acid and citric acid were closely related to acidity formation (Gao et al., 2018b), with the former having no significant change and the latter increasing in content. Consider that the acidity may also be related to the concentration of hydrogen ions and the concentration of the buffer. Therefore, during the development of commercial watermelon fruit, the increase of acidity may result from the comprehensive action of many factors. VOCs are closely related to aroma, we screened 11 metabolites that may be more strongly associated with flavor from 23 different VOCs. Along with them, 2-hexenal, (E)- was thought to have the scent of leaves (Bianchi et al., 2020), and unsaturated C9 aliphatic aldehydes and alcohol are considered to be the prevalent compounds in watermelon (Hatanaka et al., 1975). Besides, 5,9-undecadien-2-one,6,10- dimethyl-,(E)- and 3-buten-2-one,4-(2,6,6-trimethyl-1-cyclohexen-1-yl)- were thought to be related to the content of carotenoids (Liu et al., 2012). Our study provides a more comprehensive VOC profile of watermelon than before. Further, we could combine sensory evaluation, as in tomatoes (Tieman et al., 2017), to mine for metabolites associated with flavor, in line with consumer preferences.

Transcriptome has made progress in studies on watermelon flesh hardness, color, soluble solids, etc. (Guo et al., 2015; Zhu et al., 2017; Gao et al., 2018b; Sun et al., 2020). The transcriptional profiling analysis by K-means clustering has been applied to tomato and other crops earlier (Srivastava et al., 2010). Still, there is a lack of relevant research reports in watermelon. We obtained a total of six classes through cluster analysis, among which, the DEGs in classes 4 and 5 tended to be up-regulated. In order to confirm the reliability of the clustering results, we found that an α-galactosidase, *Cla97C04G070460*, can metabolize stachyose and raffinose into disaccharides and monosaccharides, located in class 4 (Supplementary Figure 6A; Guo et al., 2019). A *PSY* gene, *Cla97C01G008760*, belonging to class 5, was the first rate-limiting enzyme in the carotenoid pathway and showed a consistent expression pattern with the accumulation of carotenoids (Supplementary Figure 6B; Sun et al., 2018; Guo et al., 2019). Similarly, *Cla97C10G20570* is a phosphate transporter located in class 5, which is essential for carotenoid accumulation in fruits, showing a continuous accumulation pattern (Supplementary Figure 6C; Zhang et al., 2017). Besides this, we found the *LCYB* gene, *Cla97C04G070940*, a vital gene controlling carotenoid synthesis, had no significant difference, which was consistent with previous studies (Supplementary Figure 6D; Zhang et al., 2020). The results can reflect that our analysis method is effective; genes with the same trend have a regulatory effect on metabolites.

Combined transcriptome and metabolome analysis provides an important tool for the mining of metabolic networks and key genes. For example, in mulberry, the key genes regulating anthocyanins and proanthocyanidins were mined through integration analysis, and further verification showed that the abnormal expression of *bHLH3* could disrupt the homeostasis network of flavonoids and lead to differential accumulation of pigments in mulberry with different colors (Li et al., 2020). Wei et al. (2016) discovered the key genes that regulate VOCs in cucumber through integrated analysis and confirmed that functional heteromeric geranyl pyrophosphate synthase played a regulatory role in the accumulation of monoterpenes. In this study, we established the relationship between carbohydrate and glycolysis pathway genes through integration analysis, and the coexpression analysis found that five ADH genes regulate the synthesis of volatile C9 alcohols/aldehydes. ADH is an important gene involved in the conversion of alcohols and aldehydes;the expression of *CMADH3* and *CMADH12* was positively correlated with the key volatile compounds in melon (Chen et al., 2016). Our research has provided important data support for the formation of watermelon fruit flavor. Next, we will further combine biochemical experiments to verify the function of candidate genes.

## Conclusion

In summary, our results provide a global map of the transcriptome and metabolome during watermelon development. Five hundred seventeen metabolites were detected to be specifically accumulated at different developmental stages, and 344 metabolites were differentially accumulated, indicating that active metabolic activities occurred during development. Besides, genes with similar expression trends were identified by K-means clustering analysis. With the increase of sweetness and the decrease of acidity during the development process, watermelon tastes better, and 9 sugars with positive accumulation mode and 11 negative accumulation mode organic acids with negative accumulation mode are thought to play important roles. Eleven different VOCs, such as 5,9-undecadien-2-one,6,10-dimethyl- and 2,6-nonadienal, (E,Z)-, were positively accumulated during watermelon fruit development and were identified as key metabolites for watermelon aroma formation. Further, the integration of important genes in the glycolytic and LOX pathways provides molecular insights into the formation of flavor formation. In conclusion, we constructed a global map of transcriptome and metabolome changes during watermelon fruit flavor formation and provide valuable data resources for further studies on multi-omics linked with metabolomics.

## Data Availability Statement

The datasets presented in this study can be found in online repositories. The names of the repository/repositories and accession number(s) can be found below: NCBI BioProject, accession no: PRJNA703434.

## Author Contributions

WL and CG conceived and designed the experiments. WL, SZ, NH, and XL collected the watermelon accessions and participated in the material preparation. CG, WD, HZ, PY, and DY performed the experiments and analyzed the data. WL and CG wrote the manuscript. MU, MA, and MK proofread manuscripts. All authors contributed to the article and approved the submitted version.

## Conflict of Interest

The authors declare that the research was conducted in the absence of any commercial or financial relationships that could be construed as a potential conflict of interest.

## Footnotes

1 http://www.kegg.jp/

2 http://www.Plantcyc.org/

3 http://metax.genomics.cn/

4 http://cucurbitgenomics.org/organism/21

## Supplementary Material

Supplementary Figure 1 Hierarchical clustering analysis of all 443 metabolites.

Supplementary Figure 2 Correlation analysis of transcriptome data in watermelonflesh at four key developmental stages.

Supplementary Figure 3 KEGG pathway statistical analysis of differentially expressed genes (DEGs).

Supplementary Figure 4 Gene expression profile during the development of ‘Crimson’ watermelon fruit.

Supplementary Figure 5 Network representation of genes related to lipids.

Supplementary Figure 6 The FPKM of the gene has been previously reported.

Supplementary Table 1 The relative expression of 443 metabolites detected by UPLC-ESI-MS/MS of 12 samples.

Supplementary Table 2 The information of differential accumulation of metabolites detected by UPLC-ESI-MS/MS.

Supplementary Table 3 Relative expression levels of volatile organic compounds in 12 samples.

Supplementary Table 4 Differential expression of volatile organic compounds at four development stages.

Supplementary Table 5 The FPKM value of the gene was found in 12 samples.

Supplementary Table 6 The Pearson correlation coefficient between lipidS and genes.
