# Supplementary material for: Metabolome and Transcriptome Integration Reveals Insights Into Flavor Formation of ‘Crimson’ Watermelon Flesh During Fruit Development
Source: Front Plant Sci. 2021 May 12;12:629361. doi: 10.3389/fpls.2021.629361 (PMC8153042; doi:10.3389/fpls.2021.629361)
Supplement: Supplementary Figure 1 — Hierarchical clustering analysis of all 443 metabolites. [file Data_Sheet_1.pdf]

Supplementary Material

Supplementary Figures

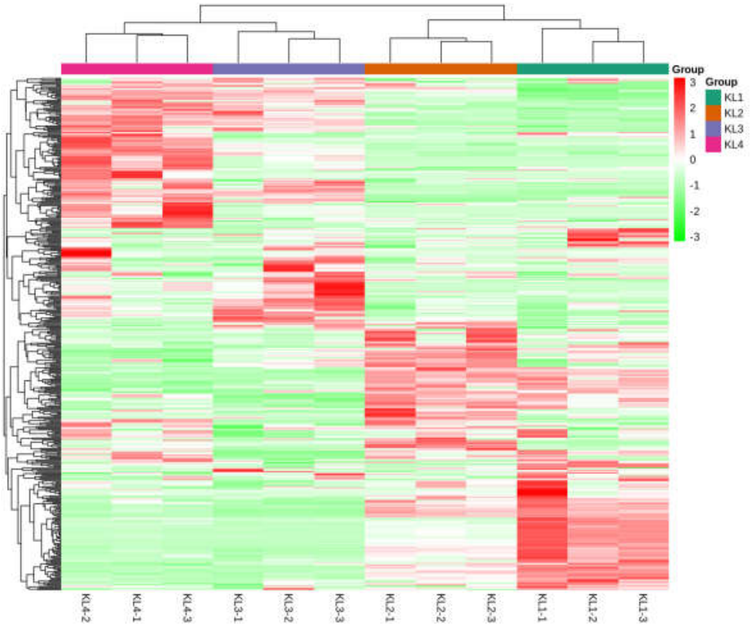

Figure S1. Hierarchical clustering analysis of all 443 metabolites detected by LC-MS. KL4. KL1, KL2, KL3, and KL4 represent the samples at 10, 18, 26, and 34 days after pollination, respectively.

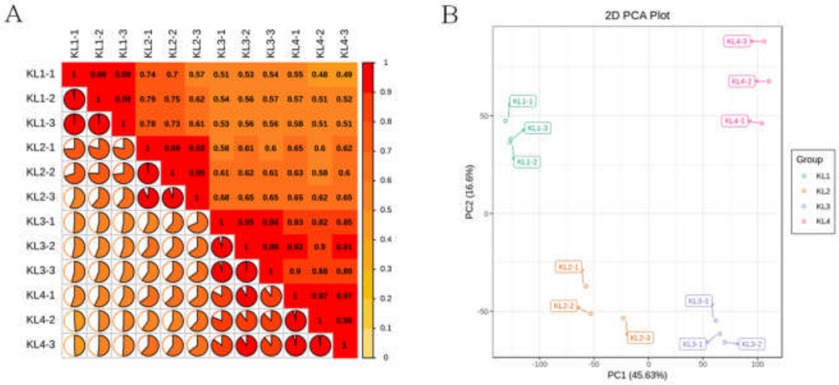

Fig. S2. Correlation analysis of transcriptome data in watermelon flesh at four key developmental stages. (A) The correlation diagram between transcriptome samples, KL1, KL2, KL3, and KL4 represent the samples at 10, 18, 26, and 34 days after pollination, -1, -2, -3 represent three biological repetitions. (B) Principal component analysis (PCA) of 12 samples, samples with the same color were three biological repeats.

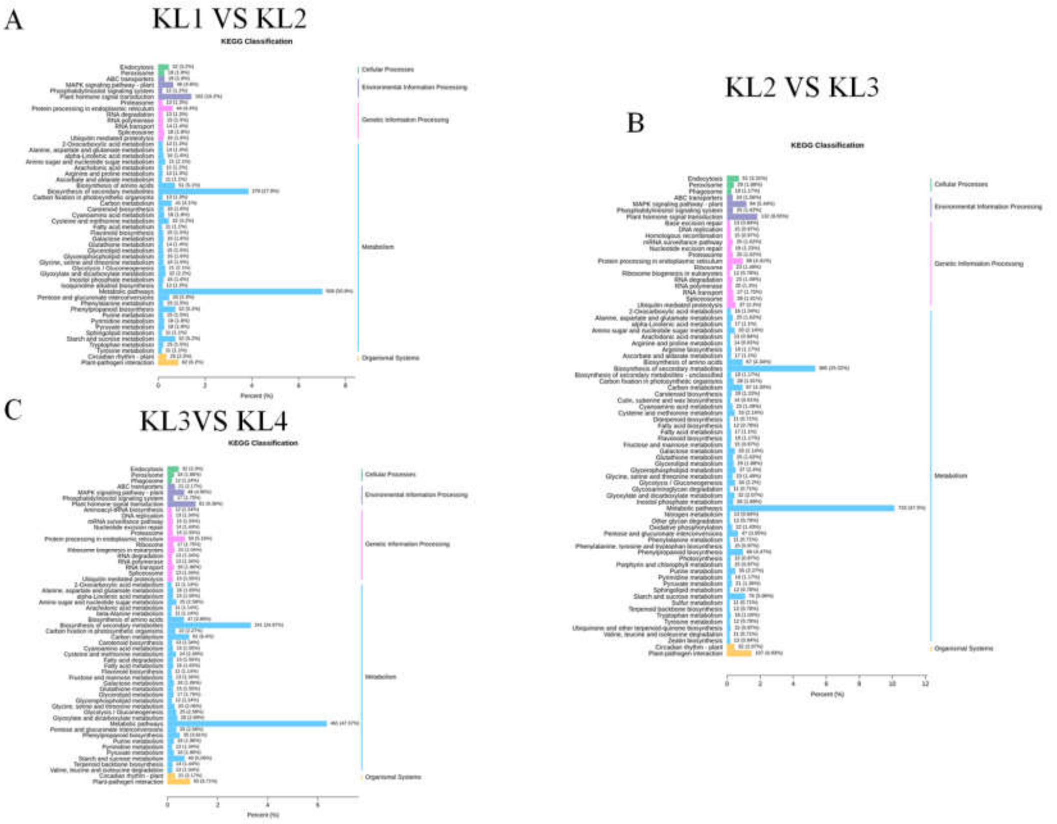

Figure S3. KEGG pathway statistical analysis of differentially expressed genes. (A)(B)(C) KEGG classification of DEMs by comparing KL1 vs. KL2, KL2 vs. KL3, and KL3 vs. KL4, Respectively. KL1, KL2, KL3, and KL4 represent the samples at 10, 18, 26, and 34 days after pollination.

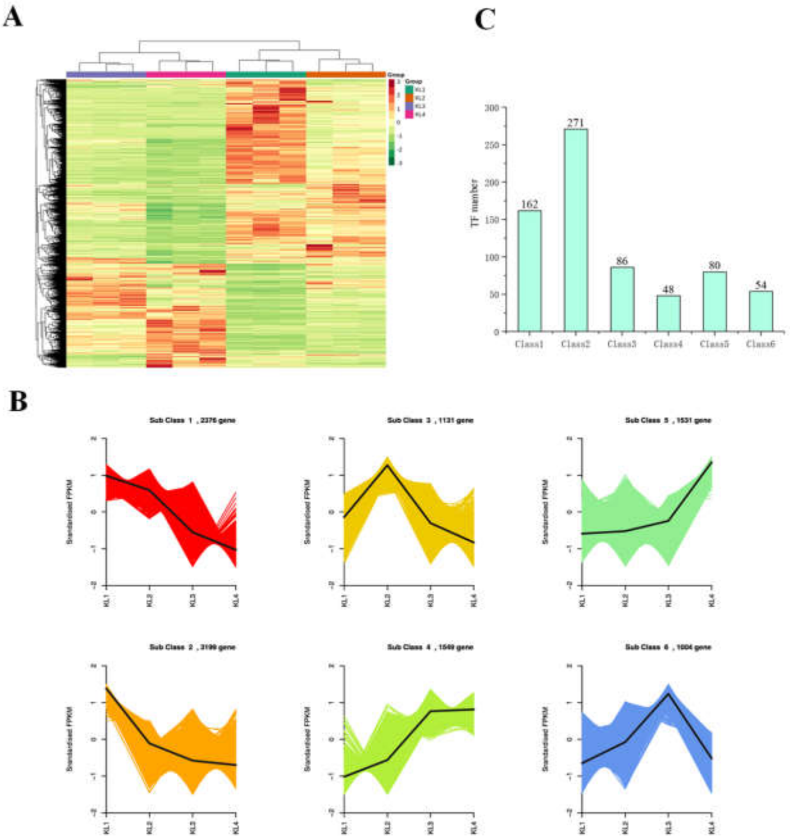

Figure S4. Gene expression profile during the development of 'Crimson'watermelon fruit. (A) Cluster heat map of all differentially expressed genes (DEGs). Each stage consists of three repetitions (B) K-means analysis of DEGs, the thick black line represents the mean value of gene expression patterns in the class, each line in the figure represents a metabolite, and different classes are shown with different colored lines. After averaging each metabolite for three times, data standardization was carried out. (C) The number of differential transcription factors identification in six classes.

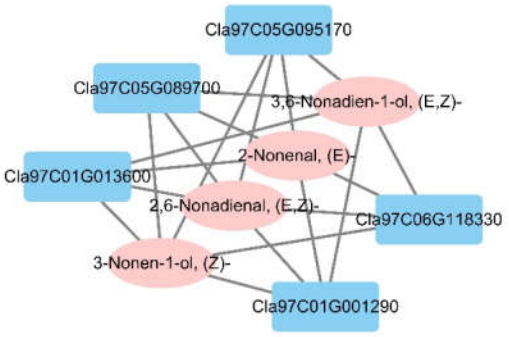

Figure S5. Network representation of genes related to lipids. Circles represent metabolites, boxes represent genes and edges represent relationships between gene and metabolite. Edges colored in red and green represent positive and negative correlations, respectively, with Pearson correlation coefficient > 0.8.

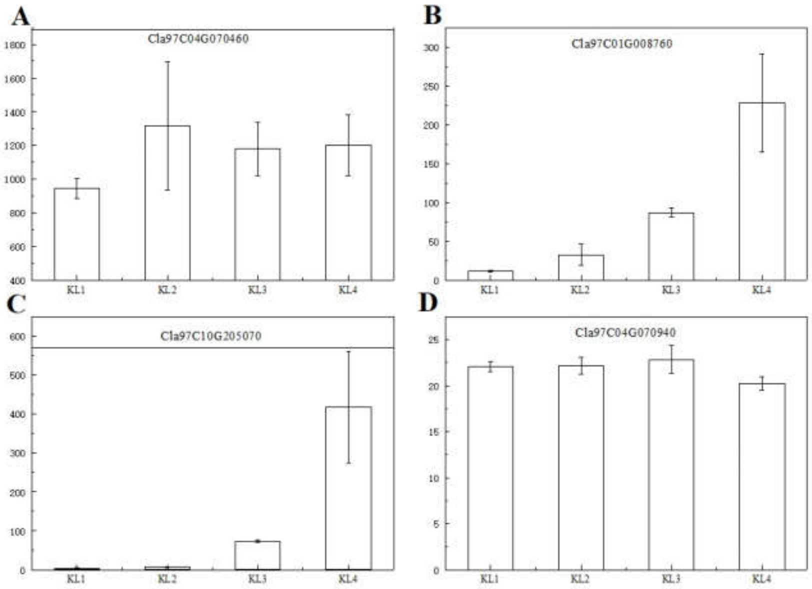

Figure S6. The FPKM of the gene has been previously reported. (A) Cla97C04G070460: α-galactosidase gene. (B) Cla97C01G008760: *PSY* gene. (C) Cla97c10g20570: phosphate transporter gene (D) Cla97C04G070940: *LCYB* gene.
